# *eClinicalMedicine*: Improving the health and wellbeing of people around the world

**DOI:** 10.1016/j.eclinm.2022.101592

**Published:** 2022-07-21

**Authors:** eClinicalMedicine

4 years ago, *eClinicalMedicine*’s first issue was published in our inaugural Editorial, we laid out our commitment to research excellence, helping to reverse inequities and inequalities in science and medicine and to serve our authors and the scientific community. Looking back now over these 5 years, we surely held our promises. We launched our Collection on Gender Equality and Health in March 2020, in order to fight gender disparities and inequality across the world and to highlight the need for change of health systems, with a multipronged focus on women. Following this collection, in June 2021, we released our two-part Collection Racial Inequity in Health which outlines and discusses racial and ethnic inequality across global settings. Coinciding with the 5-year anniversary, we received our first impact factor of 17·033, which is just one measure for excellence and quality, but as Richard Horton put it: “Impact (that) matters”; as seen in our daily devotion to science and our responsibility to the scientific community but also the whole world which become painfully apparent during the recent pandemic.

A journal is only as good as its authors and reviewers and we want to express our highest appreciation for all their help, trust, and dedication to science. With this said, we want to give the spotlight now to our authors’ and reviewers’ work and highlight five influential papers that were published in our journal over the last 4 years.

In 2018, we published the study by Amanda Sacker and colleagues, Social media use and adolescent mental health: findings from the UK Millennium Cohort Study, where the authors highlighted the detrimental effect of prolonged social media use in adolescents. They used the UK Millennium Cohort Study on 10 904 14-year-olds and found links between social media use and depression, experiences of online harassment, poor sleep, low self-esteem, and poor body image. With these data, the authors called for the development of guidelines for the safe use of social media and on the industry to regulate hours of social media use more tightly for young people.

In the following year, 2019, Yizhi Liu and colleagues published with us the Diagnostic efficacy and therapeutic decision-making capacity of an artificial intelligence platform for childhood cataracts in eye clinics: a multicentre randomized controlled trial —a study, which presented the first real-world-comparative trial testing the efficacy and accuracy of diagnostic medical Artificial Intelligence (AI) compared with clinicians. The authors randomly assigned paediatric patients without a definitive diagnosis of cataracts or history of previous eye surgery to receive a diagnosis and treatment recommendation from either the AI algorithm or senior consultants. The results showed that the accuracy of diagnosis and treatment-decision of the medical AI was lower than of the senior consultants. However, the medical AI required less time for diagnosis and still achieved a high level of patient satisfaction in eye clinics.

In 2020, Saad Omer and colleagues published their paper with us highlighting Determinants of COVID-19 vaccine acceptance in the USA. Early in the pandemic and before vaccines against COVID-19 became available, the authors undertook an online survey of 672 participants located in the USA to understand risk perceptions about the COVID-19 pandemic, acceptance of a COVID-19 vaccine, and trust in sources of information. Their survey showed that men, older adults, Asian ethnic group, and people with higher education were more likely to accept the vaccine. Overall, they found a 67% acceptance of a COVID-19 vaccine, but with demographic and geographical disparities. Based on this they recommend that the US public health officials and policymakers must prioritise effective COVID-19 vaccine-acceptance messaging, especially for those who are most vulnerable.

Going into 2021, Athena Akrami and colleagues published their study, Characterizing long COVID in an international cohort: 7 months of symptoms and their impact, in our journal. This study represents one of the first papers to systematically describe the symptoms felt by patients with long COVID. The study is based on an online survey which was created by a Patient-led Research Collaborative consisting of patients with COVID-19 in early 2020. The study included 3762 participants, from 56 countries, with illness lasting over 28 days. The authors estimated the prevalence of 203 symptoms in ten organ systems and traced 66 symptoms over seven months including systemic and cognitive symptoms. The authors concluded that given the heterogeneity of long COVID, multidisciplinary research will be necessary to understand the pathophysiology of the disease.

Now in 2022, we published the Calorie restriction improves lipid-related emerging cardiometabolic risk factors in healthy adults without obesity: Distinct influences of BMI and sex from CALERIE™ a multicentre, phase 2, randomised controlled trial by William Kraus and colleagues. The authors tested the effect of calorie restriction over two years on risk markers for atherosclerotic cardiovascular disease, insulin resistance and type 2 diabetes. 218 participants were randomized to 24 months of calorie restriction versus normal diet. The authors found that two years of approximately 12% calorie reduction improved biomarkers of cardiometabolic risk.

Looking ahead, we have already new initiatives planned. Soon we will be publishing an update of our Gender Equality and Health collection including new research on how to strengthen health systems to ensure equal health benefits across gender. Also, this year, we started our call for papers for studies focusing on obesity health-care delivery, treatments, and interventions to address weight stigma in health care. With such efforts we want to continue to publish essential, early evidence that helps researchers and clinicians to improve the health and wellbeing of people around the world.

